# Lipide: Diagnostik und Therapie bei Diabetes mellitus (Update 2026)

**DOI:** 10.1007/s00508-025-02652-1

**Published:** 2026-04-30

**Authors:** Thomas C. Wascher, Bernhard Paulweber, Hermann Toplak, Christoph H. Saely, Heinz Drexel, Bernhard Föger, Friedrich Hoppichler, Thomas Stulnig, Harald Stingl, Florian Höllerl, Martin Clodi

**Affiliations:** 1 1. Medizinische Abteilung, Mein Hanusch Krankenhaus, Wien, Österreich; 2 Universitätsklinik für Innere Medizin 1, Landeskrankenhaus Salzburg – Universitätsklinikum, Paracelsus Medizinische Privatuniversität, Salzburg, Österreich; 3 Universitätsklinik für Innere Medizin, Medizinische Universität Graz, Graz, Österreich; 4 VIVIT Institut/Abteilung für Innere Medizin I, Landeskrankenhaus Feldkirch, Feldkirch, Österreich; 5 Private Universität im Fürstentum Liechtenstein, Triesen, Liechtenstein; 6 VIVIT Institut, Akademisches Lehrkrankenaus Feldkirch, Feldkirch, Österreich; 7 Vorarlberger Landeskrankenhausbetriebsgesellschaft, Feldkirch, Österreich; 8 Abteilung für Innere Medizin, Fachklinik Schwaben, Bad Mergentheim, Deutschland; 9 Abteilung für Innere Medizin, A.ö. Krankenhaus der Barmherzigen Brüder, SIPCAN Institut, Salzburg, Österreich; 10 3. Medizinische Abteilung und Karl Landsteiner Institut für Stoffwechselkrankheiten und Nephrologie, Klinik Hietzing, Wien, Österreich; 11 Abteilung für Innere Medizin, Landesklinikum Baden-Mödling, Baden, Österreich; 12 1. Medizinische Abteilung mit Diabetologie, Endokrinologie und Nephrologie, Klinik Landstraße, Wien, Österreich; 13 1. Abteilung für Innere Medizin und Intensivmedizin, Krankenhaus der Barmherzigen Brüder Linz, Klinisches Forschungsinstitut für kardiovaskuläre und metabolische Erkrankungen, Medizinische Fakultät, Johannes Kepler Universität Linz, Linz, Österreich

**Keywords:** LDL-Cholesterin, Kardiovaskuläres Risiko, Hyperlipidämie, Therapieziele, Risikoreduktion, LDL-cholesterol, Cardiovascular risk, Hyperlipidemia, Therapeutic goals, Risk reduction

## Abstract

Hyper- und Dyslipidämie tragen maßgeblich zur kardiovaskulären Morbidität und Mortalität von Menschen mit Diabetes mellitus bei. Überzeugende Daten zeigen, dass eine medikamentöse Therapie mit Statinen, Ezetimib, PCSK9-Hemmern und Bempedoinsäure das kardiovaskuläre Risiko von Menschen mit Diabetes senken kann. Der vorliegende Artikel stellt die Behandlungsvorschläge der Österreichischen Diabetes Gesellschaft zum Einsatz lipidsenkender Medikamente dar.

## Abstract (en)

Hyperlipidemia and dyslipidemia make a substantial contribution to cardiovascular morbidity and mortality in people with diabetes mellitus. Pharmacotherapy with statins, ezetimibe, proprotein convertase subtilisin/kexin type 9 (PCSK9) inhibitors and bempedoic acid has convincingly been shown to reduce the cardiovascular risk of people with diabetes. The present article presents the treatment recommendations of the Austrian Diabetes Association for the use of lipid-lowering drugs in people with diabetes according to current scientific evidence.

## Grundsatz-Statement

Unabhängig vom Ausmaß der Hypercholesterinämie haben Menschen mit Diabetes mellitus gegenüber solchen ohne Diabetes ein deutlich erhöhtes kardiovaskuläres Risiko; kardiovaskuläre Erkrankungen sind die häufigste Todesursache bei Patienten mit Diabetes.

Aufgrund der klaren Datenlage zum kardiovaskulären Risiko bei Diabetes werden Menschen mit Diabetes mellitus in den Leitlinien der ESC überwiegend in die Risikokategorie „hohes“^1^ oder „sehr hohes“^2^ kardiovaskuläres Risiko eingeordnet. Dies gilt unabhängig vom Typ des Diabetes. Hier sei auch auf die Möglichkeit hingewiesen, den SCORE2-Diabetes-Kalkulator der ESC unterstützend anzuwenden. Entsprechend der Studienlage betonen auch die Leitlinien des American College Clinical Endocrinologists ein „extrem hohes“ Risiko für Menschen mit Diabetes mellitus mit klinisch manifester kardiovaskulärer Erkrankung.

## Lipidstatus

Folgende Parameter sind Bestandteil einer kompletten Lipiddiagnostik und sollten unbedingt erhoben werden:

- Gesamtcholesterin,
- Triglyzeride,
- HDL-Cholesterin,
- LDL-Cholesterin,
- Nicht-HDL-Cholesterin (sollte bei Triglyzeriden > 200 als Therapieziel verwendet werden),
- Lp(a) sollte einmalig bestimmt werden.

Die Bestimmung von Apolipoprotein B (ApoB) ist besonders bei Personen mit Triglyzeriden > 200 mg/dl empfohlen.

Lp(a)sollte einmal im Leben bestimmt werden, spätestens bei der Erstdiagnose eines Diabetes mellitus oder im Rahmen der kardiovaskulären Risikostratifizierung. Bei Personen mit sehr hohem Risiko und signifikant erhöhtem Lp(a) soll eine intensivere LDL-C-Senkung erwogen werden, da derzeit keine spezifische Therapie zur gezielten Senkung von Lp(a) klinisch zugelassen ist. Neue therapeutische Ansätze befinden sich aktuell in klinischen Phase-III-Outcome-Studien.

## Indikation zur medikamentösen Therapie

Grundsätzlich qualifizieren sich auf Basis der zu erreichenden Zielwerte die meisten erwachsenen Menschen mit Typ-2- und Typ-1-Diabetes für eine lipidsenkende Therapie. Unter 40-jährige Menschen mit Diabetes, die keine Diabetes-Komplikationen haben, keine weiteren Risikofaktoren und ein LDL-C < 100 mg/dl aufweisen, benötigen möglicherweise keine lipidsenkende Therapie.

## Therapieziele

Unter medikamentöser lipidsenkender Therapie sollen Lipidwerte, wie in Tab. 1 dargestellt, angestrebt werden.

**Tab. 1:** Lipidzielwerte bei mittlerem bis sehr hohem Gefäßrisiko

Zielwerte auf Basis der ESC-Leitlinie (The Task Force for the management of dyslipidaemias, Eur Heart J, 2025 [2])	
*Sehr hohes Risiko: LDL-Reduktion ≥* *50* *% des Ausgangswertes ***und*** ein LDL-Ziel <* *55* *mg/dl*	Diabetes mit manifester Atherosklerose (inklusive einem koronaren Kalziumscore > 300); Diabetes mit Endorganerkrankung (Mikroalbuminurie, Retinopathie, Neuropathie) oder zumindest mit 3 weiteren Risikofaktoren; Typ-1-Diabetes mit früher Manifestation und > 20 Jahren Dauer
*Hohes Risiko: LDL-Reduktion ≥* *50* *% des Ausgangswertes ***und*** ein LDL-Ziel <* *70* *mg/dl*	Diabetes ohne Endorganerkrankung, aber mit einer Krankheitsdauer von > 10 Jahren oder zumindest mit einem weiteren Risikofaktor
*Mittleres Risiko: LDL-Ziel <* *100* *mg/dl*	Junge Patienten (DM-1 < 35 Jahre, DM-2 < 50 Jahre) ohne weiteren Risikofaktor

Das primäre Ziel der Therapie ist das LDL-Cholesterin (Evidenzklasse A).

Ein sekundäres Therapieziel stellt besonders bei erhöhten Triglyzeriden das Nicht-HDL-Cholesterin dar (Evidenzklasse B). Erhöhte Triglyzeride sind oft ein Indikator für das Vorhandensein von erhöhtem Remnant-Cholesterin, das zumindest so atherogen, wahrscheinlich sogar atherogener ist als das LDL-Cholesterin.

## Initiale Therapie und Kombinationstherapien

Wenn keine Intoleranz oder Kontraindikation vorliegt, sollte ein Statin zur initialen Therapie herangezogen werden. Eine Fibrattherapie sollte bei sehr hohen Triglyzeriden (≥ 500 mg/dl) nach Ausschluss sekundärer Ursachen zur Senkung des Pankreatitisrisikos erwogen werden.

Als Startdosis sollte bei Statinen mit evidenzbasierten Dosierungen (zumindest 20 mg Atorvastatin oder 10 mg Rosuvastatin) begonnen werden. Ebenso besteht die Möglichkeit, initial mit einer höheren Statindosis oder aber einer Kombinationstherapie zu beginnen, wenn das Erreichen des Therapiezieles mit einer Startdosis unwahrscheinlich erscheint.

Möglichkeiten der sequenziellen Erweiterung einer Statintherapie sind bei Nicht-Erreichen des Therapiezieles und/oder Statinunverträglichkeit:

Ezetimib: (LDL‑C ca. 15 % gesenkt) und/oder Bempedoinsäure 180 mg (LDL‑C ca. 20 % reduziert).

Falls unter obiger 3‑fach-lipidsenkender Therapie die LDL-C-Zielwerte nicht erreicht werden, finden PCSK9-Inhibitoren auf Antikörperbasis Anwendung (in maximaler Dosis LDL-C > 50 % reduziert; Alirocumab 150 mg oder Evolocumab 140 mg jede zweite Woche s.c. oder Alirocumab 300 mg jede vierte Woche s.c.). Zusätzlich steht als neue Therapieoption zur Hemmung von PCSK9 Inclisiran 284 mg (alle 6 Monate s.c., LDL‑C ca. 50 % reduziert) zur Verfügung. Die zugehörige Endpunktstudie für Inclisiran ist bei Drucklegung noch im Laufen.

## Adjuvante Therapie mit Icosapentaensäureethylester

Icosapentaensäureethylester ist ein stabiler Ethylester der Eicosapentaensäure (EPA), die zur Klasse der Omega-3-Fettsäuren zählt. In der untersuchten Dosierung von 2‑mal 2 g täglich konnte – als „add-on“ zu einer vorbestehenden Therapie mit Statinen – bei Personen mit hohem oder sehr hohem kardiovaskulärem Risiko und Triglyzeriden zwischen 135 und 499 mg/dl mit und ohne Diabetes eine signifikante 25 %ige Reduktion kardiovaskulärer Endpunkte nachgewiesen werden. Keine positive Wirkung konnte im Gegensatz dazu in Studien beobachtet werden, die Omega-3-Mischpräparate aus EPA und DHA enthielten.

## Monitoring und Sicherheitslabor

Der Effekt einer eingeleiteten oder geänderten Therapie sollte nach 4 bis 6 Wochen evaluiert werden und als Basis einer etwaigen Therapieanpassung dienen. Bei stabiler Therapie sind Kontrollen zumindest alle 12 Monate anzustreben.

Laborchemische Nebenwirkungen (Muskel und Leber) sind bei Statintherapie sehr selten. CK, GOT, GPT sollten vor Beginn einer Statintherapie gemessen werden. Eine Routinekontrolle der CK im Follow-up wird bei asymptomatischen Personen nicht empfohlen.

Auf die Möglichkeit einer (sehr seltenen) symptomatischen Myopathie unter einer Therapie mit Statinen muss hingewiesen werden.

## Evidenzlage

Basis der Therapieempfehlungen sind die Leitlinien der ESC [1, 2], des AACE/ACE [3] sowie mehrere Metaanalysen der verfügbaren Statinstudien [4–6], wobei eine davon sich spezifisch auf Menschen mit Diabetes mellitus bezieht [5].

Diese Metaanalysen belegen auch den klaren Zusammenhang zwischen dem Ausmaß der LDL-C-Senkung und der Reduktion des vaskulären Risikos.

Die Evidenz zur Kombination von Ezetimib mit einem Statin stammt aus der IMPROVE-IT-Studie [7]. In dieser konnte bei Personen nach einem akuten Koronarsyndrom durch die Kombination im Vergleich zu einer Monotherapie mit Statinen eine signifikante weitere Reduktion kardiovaskulärer Ereignisse beobachtet werden.

Die Evidenz zu PCSK9-Hemmern auf Basis einer Statintherapie stammt aus der FOURIER-Studie (Evolocumab) [8] und deren Subanalysen, in denen sich Menschen mit Diabetes mellitus nicht von denen ohne Diabetes in ihren klinischen Vorteilen durch die Behandlung unterschieden. Ergänzend dazu gibt es Evidenz aus der ODYSSEY OUTCOMES-Studie (Alirocumab) [9], in der ebenfalls 29 % der Population zu Studienbeginn an Diabetes erkrankt waren.

Die Evidenz zur Therapie mit Fibraten stammt aus der VAHIT-Studie, in der eine Subgruppe von 627 Diabetikern untersucht wurde [10], sowie aus (Post-hoc‑)Analysen der FIELDS-Studie [11] und der ACCORD-Studie [12]. In den beiden Letzteren wurde Fenofibrat meist „on top“ der Statintherapie eingesetzt. Auch in der PROMINENT-Studie (10.497 Menschen mit Typ-2-Diabetes mit erhöhten Triglyzeriden [200–499 mg/dl] und niedrigem HDL‑C [≥ 40 mg/dl, Pemafibrat 2‑mal 0,2 mg täglich vs. Plazebo]) konnte kein Benefit einer Fibrattherapie gezeigt werden [13]. Die verfügbare Evidenz zeigt also keinen kardiovaskulären Benefit durch eine Triglyzeridsenkung mit Fibraten.

Die Evidenz zu Icosapentaensäureethylester stammt aus der REDUCE-IT-Studie an mit Statinen vorbehandelten Personen mit und ohne Diabetes, welche einen Triglyzeridspiegel von 135–499 mg/dl aufwiesen [14].

Die klinische Evidenz zu Bempedoinsäure stammt aus der CLEAR Outcomes-Studie [15].

Für die Kombination verschiedener Lipidsenker gibt es mit Ausnahme der oben angeführten Studien zurzeit ausschließlich die pathophysiologischen Grundlagen und epidemiologischen Daten als Evidenz.

## Footnotes

1 Ohne zusätzliche Risikofaktoren oder Endorganerkrankung.

2 Mit zusätzlichen Risikofaktoren oder Endorganerkrankung.

## Interessenkonflikt

T.C. Wascher hat von folgenden Unternehmen Forschungsunterstützungen und/oder Honorare erhalten: Amgen, AstraZeneca, MSD, Novartis, Sanofi, Daiichi-Sankyo. B. Paulweber hat von folgenden Unternehmen Forschungsunterstützungen und/oder Honorare erhalten: Amgen, AstraZeneca, Bayer, Boehringer Ingelheim, Lilly, MSD, Mylan, Novo Nordisk, Sanofi. H. Toplak hat von folgenden Unternehmen Forschungsunterstützungen und/oder Honorare erhalten: Amgen, AstraZeneca, Mylan, MSD, Novo Nordisk, Pfizer, Sanofi. C.H. Saely hat von folgenden Unternehmen Forschungsunterstützungen und/oder Honorare erhalten: Amgen, AstraZeneca, MSD, Mylan, Novartis, Sanofi. H. Drexel hat Honorare für Vorträge, Forschungsunterstützung und Beratungsaktivitäten von Novo Nordisk, Merck, MSD, Sanofi, Bayer, Amgen, Boehringer Ingelheim, Servier erhalten. B. Föger hat von folgenden Unternehmen, die teilweise auch fördernde Mitglieder der ÖDG sind, Forschungsunterstützungen und/oder Honorare erhalten: Alexion, Amgen, AOP Orphan, AstraZeneca, Böhringer Ingelheim, Janssen, Lilly, MSD, Novartis, Novo Nordisk, Sanofi, Takeda. F. Hoppichler hat von folgenden Unternehmen Forschungsunterstützungen und/oder Honorare erhalten: Amgen, Sanofi, Novartis, Daiichi-Sankyo. T. Stulnig hat von folgenden Unternehmen Forschungs‑/Reisekostenunterstützung und/oder Honorare erhalten: Alexion, Amgen, AstraZeneca, Boehringer Ingelheim, Meda, Mylan, Novo Nordisk, Sanofi. H. Stingl legt offen, dass er von folgenden Unternehmen, die teils auch fördernde Mitglieder der ÖDG sind, Forschungsunterstützungen, Honorare für Vorträge und/oder Beratertätigkeit bzw. Fortbildungsunterstützung im Rahmen der (dienst)rechtlichen Rahmenbedingungen (Einladung zu Kongressen) erhalten hat: Amgen, AstraZeneca, Boehringer Ingelheim, Eli Lilly, Gebro, Menarini, MSD, Novo Nordisk, Novartis, Sanofi Aventis, Servier, Takeda. F. Höllerl hat von folgenden Unternehmen Forschungs‑/Reisekostenunterstützung und/oder Honorare erhalten: Amarin, Amgen, Daiichi-Sankyo, Novartis, Sanofi. M. Clodi hat von folgenden Unternehmen Forschungsunterstützungen und/oder Honorare erhalten: Amgen, AstraZeneca, MSD, Pfizer, Sanofi.
